# Detection of *KRAS* G12/G13 Mutations in Cell Free-DNA by Droplet Digital PCR, Offers Prognostic Information for Patients with Advanced Non-Small Cell Lung Cancer

**DOI:** 10.3390/cells9112514

**Published:** 2020-11-20

**Authors:** Kleita Michaelidou, Chara Koutoulaki, Konstantinos Mavridis, Eleftherios Vorrias, Maria A. Papadaki, Anastasios V. Koutsopoulos, Dimitrios Mavroudis, Sofia Agelaki

**Affiliations:** 1 Laboratory of Translational Oncology, School of Medicine, University of Crete, 71110 Heraklion, Crete, Greece; mkleita@gmail.com (K.M.); xarakoutoulaki@outlook.com (C.K.); papadaki_maria1@yahoo.gr (M.A.P.); mavroudis@uoc.gr (D.M.); 2 Institute of Molecular Biology and Biotechnology, Foundation for Research and Technology-Hellas, 70013 Heraklion, Crete, Greece; mavridiskos@gmail.com; 3 Department of Medical Oncology, University General Hospital of Heraklion, 71110 Heraklion, Crete, Greece; elvorrias@gmail.com; 4 Department of Pathology, University General Hospital of Heraklion, University of Crete, Medical School, 71110 Heraklion, Crete, Greece; akoutsop@gmail.com

**Keywords:** ctDNA, NSCLC, ddPCR, liquid biopsy, prognostic biomarker, molecular testing

## Abstract

*KRAS* mutations are found in approximately one third of non-small cell lung cancer (NSCLC) patients. In this study, we aim to investigate whether *KRAS* G12/G13 mutant allele fraction (MAF) in cell-free DNA (cfDNA) can provide meaningful prognostic information in NSCLC. Multiplex droplet-digital PCR was used to quantitatively assess *KRAS* G12/G13 MAF in cfDNA from 114 pre-treated advanced disease NSCLC patients. In 14 patients, changes in *KRAS* G12/G13 MAF were longitudinally monitored during treatment. Plasma *KRAS* G12/G13 status was associated with poor patients’ outcome in terms of progression-free survival (PFS) (*p* < 0.001) and overall survival (OS) (*p* < 0.001). In multivariate analysis, the detection of plasma *KRAS* mutations was an independent predictor of adverse PFS (HR = 3.12; *p* < 0.001) and OS (HR = 2.53; *p* = 0.002). *KRAS* G12/G13 MAF at first treatment evaluation (T1) was higher (*p* = 0.013) among patients experiencing progressive disease compared to those with disease control, and increased *KRAS* MAF at T1 was associated (*p* = 0.005) with shorter PFS. On the contrary, no association was observed between tissue *KRAS* mutation status and patients’ prognosis. Our results show that ddPCR-based detection of *KRAS* G12/G13 mutations in plasma could serve as an independent biomarker of unfavorable prognosis in NSCLC patients. Changes in *KRAS* MAF can provide valuable information for monitoring patient outcome during treatment.

## 1. Introduction

In advanced NSCLC, treatment paradigms have now shifted from histology-based to genotype-based approaches [1]. Current guidelines for genetic testing include screening for the presence of activating alterations in epidermal growth factor receptor (*EGFR*), anaplastic lymphoma kinase (*ALK*), ROS proto-oncogene 1 (*ROS1*), or v-Raf murine sarcoma viral oncogene homolog B (*BRAF*) prior to the initiation of first-line treatment in NSCLC [2]. Beyond these biomarkers, Kirsten rat sarcoma viral oncogene homolog (*KRAS*) mutations, and specifically the *KRAS* G12C point mutation, will likely soon be approved as biomarkers for the selection of patients eligible for treatment with direct inhibitors such as AMG510 [3].

*KRAS* mutations, and in particular single amino acid substitutions in codon 12 (e.g., G12C, G12V, and G12D) are the most prevalent gain-of-function alterations found in 20–40% of lung adenocarcinomas [4]. These oncogenic mutations, hold the KRAS oncoprotein in a constitutively active state and are associated with the development and progression of several cancers, including NSCLC [5]. Nevertheless, the prognostic and predictive value of *KRAS* mutations in NSCLC is still not well established [6,7].

To date, the majority of studies are based on tumor tissue genotyping [7]. Although tissue biopsy is considered as the “gold standard” source for the molecular profiling of cancer, in advanced NSCLC, access to tumor tissue is often limited [8,9]. Additionally, the procedure is invasive and cannot capture the temporal and spatial heterogeneity of the tumor [9]. To this end, circulating tumor DNA (ctDNA), the fraction of circulating cell-free DNA (cfDNA) that derives from cancer cells, represents a valuable analyte within “liquid biopsy” by providing an alternative, reliable and non-invasive source of clinically valuable information [10,11]. Furthermore, ctDNA levels have been correlated with tumor load in lung cancer patients and dynamic changes have the potential to monitor tumor burden and the biological alterations of the tumor under the pressure of anticancer treatment [12].

Detection and quantification of tumor-derived mutations in cfDNA is technically challenging, considering that cfDNA molecules are highly fragmented (134–144 bp) by nature and diluted by cfDNA of non-malignant origin [11,13]. The third generation PCR technology, droplet digital PCR (ddPCR), has the potential to fulfill the need for rapid, robust and highly sensitive detection of mutations of clinical importance in cfDNA, with mutant allele frequencies (MAF) as low as 0.01% [13]. The sensitivity of ddPCR is superior compared to standard quantitative PCR assays in detecting low target copies and has a simplified workflow compared to other digital PCR technologies [8,14].

Based on the above, we hypothesized that the concentration of *KRAS* mutations in plasma cfDNA is associated with outcomes in patients with advanced NSCLC treated with chemotherapy and that monitoring of ctDNA levels may provide indications of treatment efficacy. In this study, we applied a sensitive multiplex ddPCR-based assay to detect and quantify the seven most common hotspot mutations in codons 12 and 13 (G12A, G12C, G12D, G12V, G12R, G12S, and G13D) of *KRAS* oncogene in cfDNA isolated from plasma samples obtained from NSCLC patients prior to initiating first-line treatment for recurrent or metastatic disease. Our primary goal was to investigate whether plasma *KRAS* G12/G13 MAF, both as a categorical variable and as a continuous measure, has prognostic implications in patients treated with first-line systemic therapy. An additional aim of our study was to evaluate the variation of *KRAS* G12/G13 MAF in a subset of serially acquired plasma samples from patients with baseline mutant cfDNA, to explore potential associations with response to treatment.

## 2. Materials and Methods

### 2.1. Study Population

A total of 114 whole blood samples were obtained from patients with advanced NSCLC before starting first-line systemic therapy, at the Department of Medical Oncology, University General Hospital of Heraklion, Crete, Greece, between 2017 and 2020. All patients received standard first-line regimens according to current treatment guidelines. Patients’ baseline characteristics such as age, smoking history, the Eastern Cooperative Oncology Group Performance Status, histological subtype, number of metastatic organs, TNM stage and PD-L1 status were also collected for statistical analyses. Data concerning tissue genotyping were also collected, and patients carrying *EGFR* mutations or *ALK* translocations in tumor samples were excluded from the analysis.

Follow-up data included progression-free survival (PFS) and overall survival (OS). PFS was defined as the time elapsed between the initiation of first-line treatment and first documented disease progression or death. OS was determined as the time interval from the beginning of first-line treatment to the date of death from any cause (Table 1). Response to treatment was evaluated according to the Response Evaluation Criteria in Solid Tumors (RECIST) 1.1, and described as partial response (PR), stable disease (SD), and progressive disease (PD) [15].

This study complied with the ethical standards of the World Medical Association’s Declaration of Helsinki, as revised in 2013. The research protocol was approved by the institutional ethics committee of the University Hospital of Heraklion (20/12-07-17), and all participants provided written informed consent to participate in the study.

### 2.2. Plasma Processing and Cell-Free DNA (cfDNA) Extraction

Pre-treatment peripheral blood samples were collected from all NSCLC patients in K_2_ EDTA blood collection tubes. The plasma fraction was obtained by a two-step centrifugation protocol, within 2 h upon receipt. Firstly, blood was centrifuged at 1300× *g* for 15 min at 4 °C. Then, the supernatant was collected and centrifuged at 2000× *g* for 15 min at 4 °C. Isolated plasma samples were stored at −80 °C until further processing.

Total cfDNA was extracted from 2 mL of plasma from each sample, using the QIAmp circulating nucleic acid kit (Qiagen) according to the manufacturer’s protocol. cfDNA was eluted in 60 μL of nuclease-free water and quantified using the Qubit dsDNA HS Assay kit (Life Technologies) on Qubit fluorometer 2.0 (Life Technologies). The concentration of cfDNA was expressed in ng/μL (median cfDNA concentration = 1.45 ng/μL and range 0.25–800.0 ng/μL; Table 2).

### 2.3. Blood-Based KRAS G12/G13 Mutation Analysis via Droplet Digital PCR (ddPCR)

ddPCR was performed using the QX200 Droplet Digital PCR System (Bio-Rad, Hercules, CA, USA) and the *KRAS* G12/G13 Screening Multiplex Kit (Bio-Rad). This multiplex assay can distinguish wild-type from mutant *KRAS* exon 2 sequence, but is not able to distinguish among individual mutations in codons 12/13. In particular, the seven most common mutations in *KRAS* exon 2 (G12A, G12C, G12D, G12R, G12S, G12V, and G13D) included in this multiplex assay were quantified in plasma by ddPCR.

Each ddPCR reaction mixture contained the 1X *KRAS* G12/G13 multiplex screening kit assay reagent (Bio-Rad) which includes primers and probes (wild-type probe labeled with HEX dye and mutant probe marked with FAM dye), 1X ddPCR Supermix for probes (no UTP; Bio-Rad), 5U restriction enzyme MseI (New England Biolabs), and 4 μL of cfDNA template, adjusted to a final volume of 20.0 μL with DEPC-treated water. Thereafter, samples were mixed with 70.0 μL of droplet generator oil for probes (Bio-Rad) and partitioned into up to 20,000 droplets using QX200 droplet generator (Bio-Rad). Emulsified samples were transferred on 96-well plates (Bio-Rad) and endpoint PCR was performed on a C1000 Touch thermal cycler (Bio-Rad). The following thermal cycling conditions were used, according to the manufacturer’s recommendations: 95 °C for 10 min, and 40 cycles of 94 °C for 30 s, 55 °C for 1 min, and 98 °C for 10 min. The plate was then transferred and read in the FAM and HEX channels using the QX200 droplet reader (BioRad). Each sample was tested in two technical replicates and every ddPCR run included negative template controls (NTCs) and positive template controls (PTCs) to calculate fluorescence thresholds. The PTCs used were synthetic DNA fragments for wild-type only and mutant sequence (gBlock gene fragments of ∼500 bp covering *KRAS* exon 2, Integrated DNA Technologies), and gDNA extracted from cancer cell lines with known *KRAS* G12/G13 mutation status (HT-29, wild-type; LS174T, heterozygous for G12D; A-549, homozygous for G12S).

Data analysis was achieved using the QuantaSoft Analysis Pro Software (Version 1.0.596) to assign positive and negative droplets and to provide an absolute quantification of target DNA (target copies/μL of reaction). Threshold was manually set based on positive control samples for each channel, and a cut-off of three positive droplets (Rule of three) and the limit of detection of the assay were used to call a sample *KRAS* G12/G13 mutant. Wells with less than 10,000 accepted droplets were excluded from further analyses.

*KRAS* G12/G13 mutant allele concentration (copies/µL reaction, C_MUT_) and wild-type (WT) allele concentration (copies/µL reaction, C_WT_) were used for the calculation of MAF using the equation: (C_MUT_/(C_MUT_ + C_WT_)) × 100. Moreover, since it has been reported that MAF might be influenced by variable levels of non-tumor wild-type DNA present in each sample [13], we also calculated the number of *KRAS* G12/G13 mutant copies/mL of original plasma used from each patient.

### 2.4. Determination of the Detection Limit of the ddPCR-Based KRAS G12/G13 Multiplex Assay

The limit of detection (LoD) is an important performance parameter to be established for validation of ddPCR measurements, and is defined as the lowest mutant allele fraction (MAF) that can be reliably detected and is distinguishable from the background or negative control.

For the determination of the LoD of the *KRAS* G12/G13 multiplex assay, a positive control mutant template (gBlock carrying the *KRAS* c.34G > C/p.G12R mutation) was diluted in a background of wild-type DNA (gBlock, *KRAS* exon 2 wild type sequence) to obtain a series of standard samples with the desired MAF range (50%, 10%, 1%, 0.2%, 0.1%). Additionally, 2 mL of pooled plasma from three healthy donors was spiked with the same standard samples with MAFs of 50%, 10%, 1%, 0.2%, 0.1%, and cfDNA was extracted as described above.

Each dilution point from standard samples and cfDNA extracted from spiked plasma, were analyzed in triplicate using ddPCR and the resulting data were merged during the calculation of the MAF of mutant *KRAS*.

### 2.5. Serial Monitoring of KRAS G12/G13-Mutated cfDNA in NSCLC Patients

In a subset of 14 patients with detectable *KRAS* G12/G13 mutations at baseline (T0; prior to treatment initiation), longitudinal sampling was performed at the time of first treatment response evaluation (T1; at 6–9 weeks according to treatment schedule) and on disease progression or end of first-line treatment, whichever occurred first (T2). For 6 patients, plasma samples were available at all three time points and for the remaining 8, only samples obtained at T0 and T1 were available due to disease progression at the time of first evaluation of treatment response.

At each time-point, ddPCR was carried out as described above to determine *KRAS* G12/G13 MAF and mutant copies/mL of plasma, and to monitor changes between baseline and T1 or T2 time points. Patients were divided into disease control group if they exhibited PR or SD (non-PD) and disease progression group if they experienced disease progression (PD).

### 2.6. Tissue-Based KRAS Mutational Analysis via SANGER Sequencing

In 96 out of 114 cases, *KRAS* mutation status was determined in formalin fixed paraffin embedded (FFPE) tissues using standard testing via Sanger sequencing and genomic DNA extracted from manually microdissected tumor cells. For the remaining 18 cases, *KRAS* genotyping data were not available due to inadequate tissue sample for further molecular analyses.

For Sanger sequencing, specific primers were designed to flank the *KRAS* exon 2 region which includes the hotspot mutations in codons 12 and 13. The primer sequences were as follows: *KRAS* Forward: 5′-TAAGGCCTGCTGAAAATGAC-3′ and *KRAS* Reverse: 5′-GTCCTGCACCAGTAATATGC-3′, resulting in a PCR amplicon of 165 bp.

PCR products were purified with the Nucleospin PCR clean-up kit (Macherey-Nagel), according to the manufacturer’s protocol. Cycle sequencing analysis was done in a final volume of 10.0 μL using: 2.0 μL of purified PCR product, BigDye Terminator v3.1 Sequencing reagents (Applied Biosystems, Foster City, CA, USA), and 125 nM each of the forward and reverse primers. The cycling conditions were as follows: 96 °C for 2 min, followed by 25 cycles of 96 °C for 10 s, 50 °C for 5 s, and 60 °C for 2 min. The reaction products were resolved by capillary electrophoresis on an ABI 3130 Genetic Analyzer (Applied Biosystems). Electropherograms were screened for gene alterations using the Sequencing Analysis software v5.4 (Applied Biosystems) and aligned using NCBI’s BLAST algorithm (https://blast.ncbi.nlm.nih.gov/Blast.cgi).

### 2.7. Statistical Analysis

*KRAS* G12/G13 mutation load in cfDNA (MAF and copies/mL plasma) as assessed by ddPCR and patients’ clinical features and follow-up data, were subjected to statistical analyses using the IBM SPSS Version 23 software. *KRAS* G12/G13 mutation load was analyzed as both a categorical (presence versus absence of *KRAS* G12/G13 mutation in cfDNA) and continuous measure (log-transformed values of MAF, and *KRAS* G12/G13 mutant copies/mL). The association between *KRAS* G12/G13 plasma mutation status and clinicopathological features was examined by the chi-squared test or Fisher’s exact test. The Mann–Whitney U, Jonckheere–Terpstra and Kruskal-Wallis tests were employed to scrutinize the differences of *KRAS* G12/G13 MAF between distinct groups of NSCLC samples, as appropriate.

Survival analysis was performed by constructing Kaplan–Meier PFS and OS curves and significance was evaluated using the log-rank test. Univariate and multivariable Cox proportional hazards models were constructed for the endpoint of interest, in order to determine the prognostic value of *KRAS* G12/G13 mutation load and other clinical parameters. The full multivariate regression model was adjusted for important clinical factors and currently used strong prognostic indicators for NSCLC, including TNM stage, number of metastatic organs at diagnosis and performance status. Hazard ratios (HR) and 95% confidence intervals (CI) were also calculated.

For longitudinal samples, additional analyses were performed to evaluate whether variation of *KRAS* G12/G13 MAF and mutant copies/mL in cfDNA is associated with disease progression during treatment in NSCLC patients. In more detail, we calculated the *KRAS* G12/G13 MAF in each time point, and thereafter the MAF ratio (T1/T0 and T2/T0) to investigate the fold change of mutational load from baseline. An arbitrary cut-off of ≥1.5-fold change from baseline in *KRAS* G12/G13 MAF was used to dichotomize our patient cohort into *KRAS* G12/G13 MAF-increase group and *KRAS* G12/G13 MAF-steady/decrease group. Similar analysis was also performed for *KRAS* G12/G13 mutant copies/mL and the same cut-off of ≥1.5-fold change from baseline was used to dichotomize our patient cohort. The impact of *KRAS* G12/G13 mutational load change from baseline was assessed using the Kaplan–Meier method. The Mann–Whitney U test was used to compare *KRAS* G12/G13 MAF in the disease control (non-PD) and disease progression (PD) groups.

The Cohen’s Kappa coefficient (k) was used to measure the level of inter-technique agreement of *KRAS* mutation detection between plasma and paired tissue samples.

For all statistical analyses, *p* values less than 0.05 were regarded as statistically significant.

## 3. Results

### 3.1. Patients’ Characteristics

A total of 114 cases with recurrent or metastatic NSCLC were included in this study. Most patients (75.44%) received first-line platinum-based chemotherapy. Among 96 patients with available *KRAS* tissue genotyping data: 35 (36.46%) carried a *KRAS* codon 12/13 mutation in tumor tissue and 61 (63.54%) were *KRAS* codon 12/13 wild-type. The frequency of detection of specific *KRAS* mutations among mutated samples was as follows: G12C, 45.71% (N = 16/35); G12D, 28.57% (N = 10/35), G12V, 14.29% (N = 5/35), G13D, 5.71% (N = 2/35), G12S, 2.86% (N = 1/35) and G12A, 2.86% (N = 1/35). These mutations are all included in the ddPCR *KRAS* G12/G13 multiplex kit used for plasma-based analyses. Patients’ characteristics are summarized in Table 1.

### 3.2. Determination of the Limit of Detection of the ddPCR KRAS G12/G13 Multiplex Assay

Before applying ddPCR to detect *KRAS* G12/G13 mutations in cfDNA of patient samples, we determined the LoD of the assay using standard DNA samples with a MAF range of 50%, 10%, 1%, 0.2% and 0.1% and cfDNA extracted from spiked plasma using the same standards with the desired MAFs. The observed MAFs using ddPCR were, for the standard samples, 53.10%, 10.67%, 1.06%, 0.34% and 0.0%, respectively, and for the spiked samples, 56.10%, 10.62%, 1.26%, 0.39%, and 0.16%, respectively. The linearity range of 0.2% to 50% (concordance correlation coefficient: R^2^ = 0.997 for standards and R^2^ = 0.991 for spiked standards) indicates that samples with MAF levels higher than 0.2% can be reliably quantified and steadily detected with the ddPCR *KRAS* G12/G13 multiplex assay. Mutant alleles at a frequency of 0.1% were not consistently detected, and therefore the LoD of this *KRAS* ddPCR kit was defined as 0.2%, equivalent to two mutant copies per 1000 copies of DNA input and in agreement with the reported assay sensitivity [8].

### 3.3. Detection of KRAS G12/G13 Mutations in Plasma Cell-Free DNA via ddPCR: Association with Patients’ Clinicopathological Features and Response to Treatment

Plasma *KRAS* G12/G13 mutational load was analyzed quantitively in 114 plasma samples using the multiplex ddPCR assay. Twenty-seven (23.68%) out of 114 patients were carriers of *KRAS* G12/G13 mutations in plasma cfDNA. In these patients, the median plasma *KRAS* G12/G13 MAF was 3.95% (range, 0.20–28.27%) and the median *KRAS* mutant copies/mL of plasma were 192.0 copies/mL (range, 19.70–18,600.0 copies/mL). The remaining 87 patients (76.32%) were plasma *KRAS* G12/G13 wild-type (Table 2).

We further investigated potential associations between *KRAS* G12/G13 mutation status in cfDNA with the clinicopathological parameters of patients. In our cohort, *KRAS* G12/G13 cfDNA status was negatively associated (*p* = 0.031) with disease control during treatment, since *KRAS* mutations were detected in 37.50% of patients experiencing stable disease (SD) or partial response (PR) as compared to 62.50% of patients presenting progressive disease (PD) at first disease evaluation. *KRAS* cfDNA mutation status was also associated with the presence of bone metastases (*p* = 0.022) but not with liver (*p* = 0.844) or brain metastases (*p* = 0.899). No other statistically significant associations were observed between *KRAS* G12/G13 MAF in cfDNA and TNM stage (*p* = 0.213), smoking status (*p* = 0.135), PD-L1 status (*p* = 0.239), histologic subtype (*p* = 0.183), performance status (*p* = 0.379), gender (*p* = 0.361), and number of metastatic organs at diagnosis (*p* = 0.064) (Table 3).

### 3.4. KRAS G12/G13 Mutation Status in Plasma is Associated with Poor PFS and OS in NSCLC Patients

**Figure 1:**
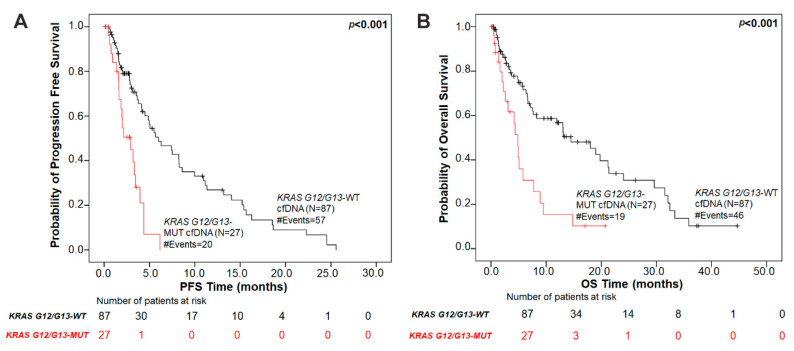
Association of plasma *KRAS* G12/G13 mutation status with prognosis in NSCLC patients. Kaplan–Meier survival curves of (**A**) progression-free survival (PFS) and (**B**) overall survival (OS) based on *KRAS* G12/G13 mutation status in plasma. *p* values were calculated via the log-rank algorithm. The median PFS of patients with *KRAS* G12/G13-mutant cfDNA was 2.93 months as compared to 5.98 months for patients in the *KRAS* G12/G13-wild type cfDNA group (*p* < 0.001). The median OS duration for patients with *KRAS* G12/G13 mutations in plasma was 4.87 months versus 14.50 months for *KRAS* G12/G13-wild type plasma group (*p* < 0.001).

Next, we performed Kaplan–Meier survival analysis to investigate whether *KRAS* G12/G13 mutation status in cfDNA provides meaningful prognostic information in patients with NSCLC. Firstly, *KRAS* G12/G13 mutational load in plasma was used as a categorical variable (presence versus absence of *KRAS* G12/G13 mutations in cfDNA) to dichotomize our patient cohort into two groups. NSCLC patients in the *KRAS* G12/G13-mutant cfDNA group had significantly (*p* < 0.001) shorter PFS intervals compared to patients in the *KRAS* G12/G13-wild type cfDNA group (Figure 1). The median PFS of patients with *KRAS* G12/G13-mutant cfDNA was 2.93 months (95% CI = 1.61–6.15 months) as compared to 5.98 months (95% CI = 4.14–25.58 months) for patients in the *KRAS* G12/G13-wild type cfDNA group.

Regarding OS, NSCLC patients harboring *KRAS* G12/G13-mutant cfDNA exhibited a significantly (*p* < 0.001) inferior OS compared with patients in the *KRAS* G12/G13-wild type cfDNA group (Figure 1). The median OS duration was 4.87 months (95% CI = 2.24–7.73 months) and 14.50 months (95% CI = 7.50–21.37 months), respectively.

Using univariate Cox regression analysis, we further confirmed that *KRAS* G12/G13 mutation status in cfDNA is a prognostic indicator of unfavorable PFS and OS in NSCLC patients. In more details, NSCLC patients with *KRAS* G12/G13-mutant cfDNA had significantly higher risk of relapse ((HR) = 3.42, (95% CI) = 1.92–6.07, *p* < 0.001) and significantly increased risk of death ((HR) = 2.72, (95% CI) = 1.55–4.77, *p* < 0.001) over time, compared to the patients in the *KRAS* G12/G13-wild type cfDNA group.

Furthermore, univariate analysis using *KRAS* G12/G13 mutational load in cfDNA as a continuous variable (log-transformed values of MAF and *KRAS* G12/G13 mutant copies/mL of plasma) corroborated the above-mentioned results. As shown in Table 4, the risk of relapse ((HR) = 1.97, (95% CI) = 1.25–3.11, *p* = 0.003) and death ((HR) = 2.04, (95% CI) = 1.28–3.24, *p* = 0.003) was significantly associated with *KRAS* G12/G13 MAF in cfDNA. Similarly, the risk of relapse ((HR) = 1.76, (95% CI) = 1.26–2.47, *p* = 0.001) and death ((HR) = 1.65, (95% CI) = 1.18–2.30, *p* = 0.003) was also significantly associated with *KRAS* G12/G13 mutant copies/mL of plasma.

Additional clinicopathological variables associated with poor patients’ prognosis included TNM stage, number of metastatic organs and performance status (All HR > 1.0, and *p* values < 0.05; Table 4).

### 3.5. Plasma KRAS G12/G13 MAF Independently Predicts for Unfavorable Prognosis in NSCLC

A multivariate Cox regression analysis was performed to evaluate whether *KRAS* G12/G13 mutations in plasma cfDNA bare independent prognostic implications in terms of both PFS and OS. We firstly developed a full multivariate model that included all statistically significant predictors detected in the univariate analysis (Table 5). In this multivariate model, the presence of plasma *KRAS* G12/G13 mutations, was found to be a strong independent predictor of adverse prognosis for both PFS ((HR) = 3.12, (95% CI) = 1.72–5.67, *p* < 0.001) and OS ((HR) = 2.53, (95% CI) = 1.40–4.56, *p* = 0.002) in NSCLC patients. We then repeated this analysis using *KRAS* G12/G13 MAF in cfDNA as a continuous variable. *KRAS* G12/G13 MAF in cfDNA retained its independent unfavorable prognostic nature with an HR of 1.99 for PFS ((95% CI) = 1.25–3.16, *p* = 0.004) and an HR of 1.85 ((95% CI) = 1.21–2.85, *p* = 0.005) for OS.

In the second step, separate reduced multivariate models were developed for plasma *KRAS* G12/G13 mutational load and each of the variables used in the full model. Interestingly, this analysis showed that the impact of *KRAS* G12/G13 mutations in ctDNA, both as a binary and as a continuous variable, on patients’ outcome is independent from TNM stage (PFS: (HR) = 3.05, (95% CI) = 1.69–5.48, *p* < 0.001; OS: (HR) = 2.70, (95% CI) = 1.51–4.82, *p* = 0.001 as a dichotomous variable; PFS: (HR) = 1.82, (95% CI) = 1.16–2.86, *p* = 0.009; OS: (HR) = 1.93, (95% CI) = 1.23–3.03, *p* = 0.004 as a continuous variable), number of metastatic organs (PFS: (HR) = 3.20, (95% CI) = 1.79–5.73, *p* < 0.001; OS: (HR) = 2.48, (95% CI) = 1.40–4.39, *p* = 0.002 as a dichotomous variable; PFS: (HR) = 1.92, (95% CI) = 1.22–3.04, *p* = 0.005; OS: (HR) = 1.95, (95% CI) = 1.23–3.08, *p* = 0.004 as a continuous variable) and performance status (PFS: (HR) = 3.55, (95% CI) = 1.98–6.36, *p* < 0.001; OS: (HR) = 2.73, (95% CI) = 1.55–4.80, *p* =0.001 as a dichotomous variable; PFS: (HR) = 2.08, (95% CI) = 1.32–3.28, *p* = 0.002; OS: (HR) = 1.89, (95% CI) = 1.21–2.95, *p* = 0.005 as a continuous variable); Table 5.

### 3.6. Concordance between the Detection of KRAS G12/G13 Mutations in Plasma and Tissue Samples

In the next step of our study, we examined the concordance in *KRAS* G12/G13 mutation status as determined using ddPCR in plasma cfDNA and via Sanger sequencing in tissue samples. Among 35 patients with *KRAS* G12/G13 mutant tumor samples, 18 patients (51.43%) carried *KRAS* mutations in plasma and 17 (48.57%) were cfDNA *KRAS* wild-type. When different types of *KRAS* mutations in tissue were considered, the frequency of mutation detection in plasma was as follows: 37.50% (N = 6/16) for G12C, 40.0% (N = 4/10) for G12D, 80% (N = 4/5) for G12V, 100% (N = 2/2) for G13D and 100% (N = 1/1) for G12S and G12A.

Of the 96 NSCLC sample pairs analyzed, we found that 18 patients (18.75%) were positively concordant; that is, *KRAS* G12/G13 mutations were detected in both cfDNA by ddPCR and in tumor tissues via Sanger sequencing. Additionally, 54 patients (56.25%) were negatively concordant; that is, both plasma cfDNA samples and tumor DNA were *KRAS* G12/G13 wild-type. These results yield an overall inter-assay concordance in *KRAS* mutation detection of 75.0% (concordance in 72/96 paired samples). The Cohen’s kappa coefficient was 0.395 (SE, 0.098; 95% CI, 0.20–0.59; *p* < 0.001), suggesting a moderate agreement between the two methods. For the remaining 24 patients, results between ddPCR in plasma and Sanger sequencing in tissue were discordant. In more detail0, *KRAS* mutations were not identified in the plasma of 17 (17.71%) patients with *KRAS* mutant tumor tissue, whereas no mutations were detected in the tumor tissue of 7 (7.29%) patients with *KRAS* mutant ctDNA ( Table S1).

### 3.7. Serial Monitoring of KRAS G12/G13 Mutations in Plasma cfDNA

Τhe impact of variations of *KRAS* G12/G13 mutation load in cfDNA in the monitoring of tumor burden was investigated in a subset of 14 NSCLC patients with detectable plasma *KRAS* G12/G13 mutations at baseline (T0).

**Figure 2:**
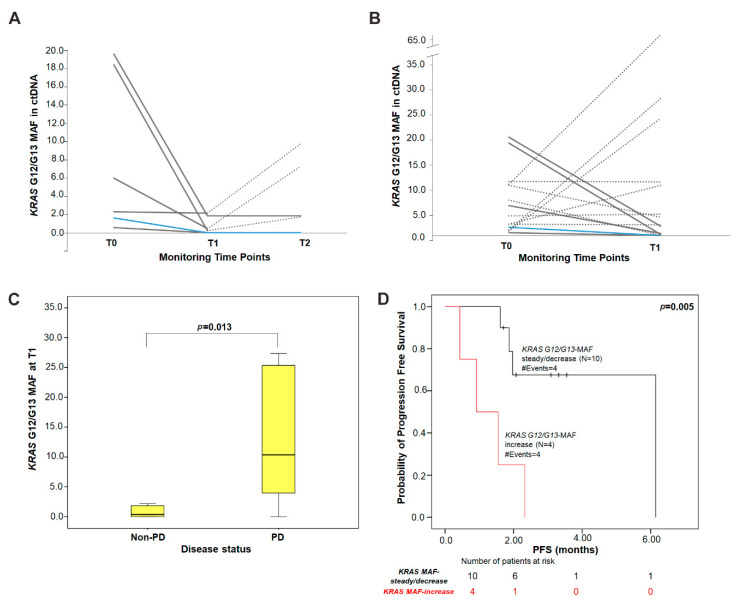
Serial monitoring of *KRAS* G12/G13 MAF in plasma cfDNA during treatment. Changes in *KRAS* G12/G13 MAF in (**A**) a subset of patients (N = 6) with available three time-points during treatment and (**B**) in all patients (N = 14) from baseline to T1. Each line represents one patient; dashed lines depict progressive disease (PD); solid grey lines indicate stable disease (SD) and the blue line indicates a patient with partial response (PR); (**C**) distribution of the absolute levels of *KRAS* G12/G13 MAF at T1 in patients experiencing PD (median = 10.38% MAF) compared to patients with non-PD (PR/SD; median = 0.35% MAF). *p*-value was calculated by the Mann–Whitney U-test; (**D**) Kaplan–Meier PFS survival curve according to *KRAS* G12/G13 MAF variation from baseline to T1 (*p* = 0.005). Patients were categorized into *KRAS* G12/G13 MAF-increase group and *KRAS* G12/G13 MAF-steady/decrease group according to the cut-off of ≥1.5-fold change in MAF from baseline. *p* values were calculated via the log-rank algorithm. T0: baseline; T1: time of first response evaluation; T2: disease progression or end of first-line treatment; PD: progressive disease; PR: partial response; SD: stable disease.

Using an arbitrary cut-off of 1.5-fold change from T0, *KRAS* G12/G13 MAF decreased in 7/14 patients, remained stable in 3/14 and increased in 4/14 at the time of the first response evaluation (T1). Among 10 patients with either decreased or stable *KRAS* G12/G13 MAF, 6 experienced disease control (5/10, SD; 1/10, PR) and 4 had progressive disease (4/10, PD), whereas all 4 patients presenting an increase in *KRAS* G12/G13 MAF experienced PD at T1. Of the six patients assessable at T2, one patient presented an increase and five had steady or decreased *KRAS* G12/G13 MAF as compared to T0. The patient with increased *KRAS* G12/G13 MAF had PD, whereas two out of five patients with steady or decreased MAF had disease control (1, SD; 1, PR) and three progressed (Figure 2).

Furthermore, the absolute levels of *KRAS* G12/G13 MAF at T1 tended to be higher (*p* = 0.013; Mann–Whitney U test) in patients experiencing PD (median = 10.38%) compared to patients with disease control (non-PD; median = 0.35%) (Figure 2). Although a similar trend was observed for *KRAS* G12/G13 MAF at T2, this was not statistically significant (*p* = 0.533; median: 4.51% (PD) versus 0.93% (non-PD).

Kaplan–Meier analysis showed that patients classified in the *KRAS* G12/G13 MAF-increase group at T1 had shorter PFS intervals (median: 0.92 months; *p* = 0.005) when compared with patients in the *KRAS* G12/G13 MAF-steady/decrease group (median: 6.15 months) (Figure 2). In contrast, this did not reach statistical significance (*p* = 0.774) at T2. The same conclusions were drawn when *KRAS* G12/G13 copies/mL of plasma were used as a dichotomous variable (T1, *p* = 0.084; T2, *p* = 0.774).

### 3.8. Association of Tissue KRAS G12/G13 Mutation Status with Patient Prognosis

In our patients’ cohort, no significant relationships were observed between *KRAS* tumor mutation status and smoking history (*p* = 0.508), number of metastatic organs (*p* = 0.226), histologic subtype (*p* = 0.765), performance status (*p* = 0.340), gender (*p* = 0.791) and PD-L1 status (*p* = 0.214). The only positive association was found between *KRAS* tissue mutation status and TNM stage (*p* = 0.026), since *KRAS* mutations were more frequently found in stage IV (66.70%) as compared to stage III (33.30%) tumors.

**Figure 3:**
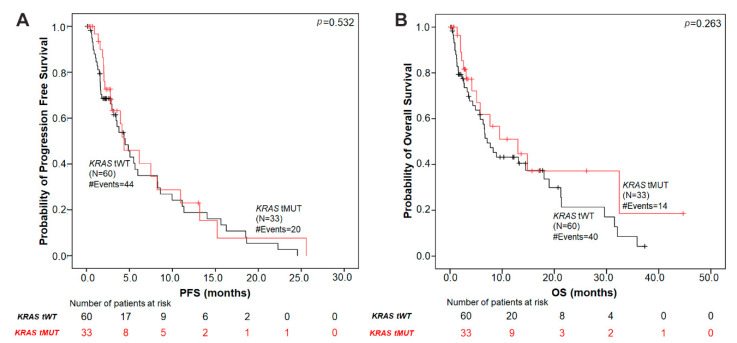
Kaplan–Meier analysis in NSCLC patients according to tumor tissue *KRAS*-mutation status. (**A**) PFS by *KRAS* G12/G13 mutation status in tumor tissue. The median PFS in patients with tissue *KRAS* mutations was 4.37 months versus 4.50 months in the *KRAS* tissue wild-type group (*p* = 0.532). (**B**) OS according to tissue *KRAS* mutation status. The median OS in tissue *KRAS* mutant patients was 13.02 months versus 7.13 months in the *KRAS* tissue wild-type group (*p* = 0.263). *p* values were calculated *via* the log-rank algorithm. tMUT, tissue *KRAS* mutant; tWT, tissue *KRAS* wild-type.

Using Kaplan–Meier survival analyses, no significant association was found between the presence of *KRAS* mutations in tumor tissue and patient’s prognosis in terms of both PFS (*p* = 0.532) and OS (*p* = 0.263) (Figure 3).

Since the most frequently observed *KRAS* mutation was G12C, we evaluated patient outcomes according to the detection of this mutation in tumor tissue. No significant difference in terms of PFS (*p* = 0.406) or OS (*p* = 0.117) was observed between patients with *KRAS* G12C mutation subtype as compared to those with *KRAS* non-G12C mutation subtypes or with *KRAS* wild-type NSCLC (Figure S1). Similarly, among patients with *KRAS* mutant tumor tissue (N = 35), no difference in PFS (*p* = 0.297) was observed between those harboring *KRAS* G12C and *KRAS* non-G12C tumor mutations (*p* = 0.297). However, patients with the *KRAS* G12C mutation had a trend for longer OS (*p* = 0.049) but not for PFS (*p* = 0.217), compared to those with *KRAS* non-G12C mutation subtypes.

### 3.9. Survival Analysis According to KRAS G12/G13 Mutational Status in cfDNA and Paired tisSue Samples

Patients with available data (N = 96) were divided into four subgroups according to *KRAS* G12/G13 mutational status in cfDNA and paired tissue samples: (1) pMUT-tMUT patients carrying mutations in both plasma and tissue, (2) pMUT-tWT patients carrying mutations only in plasma, (3) pWT-tMUT patients harboring mutations only in tissue and (4) pWT-tWT patients that were *KRAS* G12/G13 wild type in both samples. The median PFS was 1.64 months (95% CI = 0.46–4.37 months) for patients with plasma *KRAS* mutations only (pMUT-tWT) and 10.95 months (95% CI = 2.79–25.58 months) in patients with tumor tissue mutations only (pWT-tMUT). Accordingly, the median OS was 1.64 months (95% CI = 0.46–8.91 months) for pMUT-tWT and 32.45 months (95% CI = 7.66–32.45 months) for patients with *KRAS* mutations in tumor tissue only. Kaplan–Meier curves for PFS and OS according to plasma and tissue mutation status are shown in Supplementary Figure S2.

The subgroup of patients with *KRAS* mutations detected in both plasma and matched tumor tissue (pMUT-tMUT) showed shorter PFS (*p* = 0.042) and a trend for shorter OS (*p* = 0.062) compared to patients with single specimen mutations (pMUT-tWT and pWT-tMUT) and patients that were *KRAS* G12/G13 wild type in both samples (pWT-tWT) (Figure S2).

## 4. Discussion

Overall, data concerning the prognostic and predictive role of *KRAS* mutations in cfDNA isolated from patients with NSCLC remain controversial, due to the relatively small number of studies performed to date [6]. In the present study, we applied a multiplex ddPCR-based assay to detect and quantify the seven most common hotspot mutations in codons 12 and 13 (G12A, G12C, G12D, G12V, G12R, G12S, and G13D) of *KRAS* in cfDNA from a large cohort of NSCLC patients, to explore the prognostic impact of *KRAS* G12/G13 MAF prior to first-line treatment for recurrent or metastatic disease. We used ddPCR, because it allows absolute quantification of tumor-derived mutations in cfDNA with superior sensitivity compared to conventional standard methods. The limit of detection (LoD) of the multiplex ddPCR assay, used in this study, was determined to be equal to 0.2%, which is in agreement with the reported assay sensitivity, demonstrating its applicability for rapid, robust and highly sensitive quantification of *KRAS* G12/G13 mutations in NSCLC patients’ plasma samples.

Our data demonstrate that plasma *KRAS* G12/G13 MAF is associated with unfavorable PFS ((HR) = 3.42, *p* < 0.001), and inferior OS ((HR) = 2.72, *p* < 0.001) in NSCLC patients. The same results were observed when *KRAS* G12/G13 MAF in cfDNA was expressed as a continuous variable ((HR) = 1.97, *p* = 0.003 for PFS; (HR) = 2.04; *p* = 0.003 for OS). Most importantly, we show here that *KRAS* G12/G13 MAF in plasma is an independent prognosticator of unfavorable outcome with respect to PFS ((HR) = 3.12, *p* < 0.001, as a binary variable; (HR) = 1.99, *p* = 0.004 as a continuous variable) and OS ((HR) = 2.53, *p* = 0.002, as a dichotomous variable; (HR) = 1.85, *p* = 0.005 as a continuous variable), irrespective of TNM stage, number of organs affected and patients’ performance status.

A number of previously published studies showed that the detection of *KRAS* G12/G13 mutations in cfDNA are negatively associated with survival in NSCLC patients, whereas other studies did not report such a correlation. Thus, the clinical significance of *KRAS* mutations detected in cfDNA has not been fully elucidated as of yet [6]. In particular, Nygaard et al. [16], using amplification refractory mutation system-quantitative PCR (ARMS-qPCR) to detect seven *KRAS* mutations in plasma samples from advanced NSCLC, showed the independent negative prognostic value of plasma *KRAS* mutations for OS, but not for PFS. Similarly, Gautschi et al. [17], using RFLP–PCR in combination with Sanger sequencing provided evidence that the detection of plasma *KRAS* mutations is associated with poor OS. In the same study, *KRAS* mutations were detected in only 9% of plasma samples (N = 16/175 patients), whereas the study cohort was heterogenous, including patients with both early and advanced disease stages [17]. On the contrary, the study by Camps et al., performed in patients’ serum [18] and plasma [19], did not show significant differences between *KRAS* wild-type and *KRAS* mutant patients, in terms of PFS and OS. This could be attributed to the fact that only two *KRAS* mutations at codon 12 (G12C and G12V) were examined [19].

In our study, we have used the state of the art ultrasensitive ddPCR-multiplex assay, which has been repeatedly reported to have superior sensitivity compared to standard quantitative PCR assays such as ARMS-qPCR and RFLP–PCR [13,14,20], and at the same time, enabled us to perform statistical analyses using *KRAS* G12/G13 MAF in ctDNA both as a categorical and as a continuous measure, thus permitting a more reliable measurement of the mutational load and its correlations with patient outcome.

We further evaluated the clinical significance of the variation in *KRAS* G12/G13 MAF in cfDNA in a subset of 14 NSCLC patients receiving systemic first-line therapy at two subsequent time points, at the time of treatment evaluation (T1) and on disease progression (T2). Notably, the absolute levels of *KRAS* G12/G13 MAF at T1 tended to be higher in patients experiencing disease progression compared to patients with objective response or stable disease. Survival analysis at T1 showed that patients classified in the *KRAS* G12/G13 MAF-increase group had significantly shorter PFS (*p* = 0.005) when compared with patients in the *KRAS* G12/G13 MAF-steady/decrease group. Although the number of patients used for longitudinal monitoring was rather small, our data are in agreement with the results reported by Guibert et al. [21]. In their study, by using ddPCR, they showed that in 16 patients treated with chemotherapy or targeted therapy, the variation of *KRAS* mutated ctDNA correlated with the response to treatment. Finally, a recent study by Zulato et al. [22] demonstrated in a larger cohort of 58 patients with advanced disease carrying *KRAS* mutations in tumor tissue that increasing *KRAS* MAF in plasma during treatment was associated with increased probability of PD and conferred a negative impact on both PFS and OS.

We next examined the concordance in *KRAS* G12/G13 mutation status as determined using ddPCR in plasma and via Sanger sequencing in FFPE samples. The overall inter-assay concordance was found to be 75.0% (N = 72/96 paired samples; k= 0.395; *p* < 0.001), which is similar to previous studies [23,24]. In 48.6% (N = 17/35 cases) of patients with *KRAS* mutations in tumor tissues, no mutations were detected in cfDNA. Possible causes that limit the detection sensitivity of ctDNA in liquid biopsy samples include the low contribution of ctDNA originating from tumor cells into plasma, as well as the short half-life time of ctDNA in the blood circulation [9,25]. Moreover, we also found seven cases (N = 7/61, 11.5%) that were *KRAS* wild-type in tumor tissue and *KRAS* mutant in plasma. Possible causes for this discordance might include [24,26] the intra-tumoral genetic heterogeneity [25,27] and/or the presence of mutations with low fractional abundance in tissue samples falling below the detection limit of Sanger sequencing [28,29]. Unfortunately, we were not able to provide matched tumor tissue *KRAS* analysis using ddPCR in these samples, due to inadequate FFPE tissue specimens. Additionally, a false-positive plasma genotyping result due to the presence of clonal hemopoiesis [30] cannot be excluded in these patients. Overall, our study indicates the feasibility of utilizing a cfDNA assay as an alternative source to determine *KRAS* status in cases where tissue samples are unavailable or not easily accessible.

Controversial results exist regarding the prognostic value of *KRAS* mutations in lung cancer tumor tissues [31]. In contrast to the prognostic associations obtained with plasma analysis, we showed that the presence of tumor *KRAS* G12/G13 mutations in tissue was not associated with patients’ PFS or OS. Interestingly, when *KRAS* tissue status was coupled with plasma results, pMUT-tMUT patients had shorter PFS (*p* = 0.042) and a trend for shorter OS (*p* = 0.062) compared to those with single specimen mutations and the wild-type subgroups. Different *KRAS* mutation subtypes in tumor tissue have been shown to exhibit distinct associations with patient prognosis and/or therapeutic outcomes [32,33]. In the same line, we found that patients harboring the *KRAS* G12C mutation in tumor tissue had a trend for longer OS, compared to those with *KRAS* non-G12C mutation subtypes (*p* = 0.049). Analysis of a larger patient cohort using the multiplex ddPCR-based assay is required to delineate the prognostic effect of different *KRAS* mutations in NSCLC tumor tissues.

In order to better apply these study results in clinical practice, there are several potential limitations that should be cautiously considered. Firstly, although our results are in agreement with previous reports, further validation in larger patient cohorts is needed. Secondly, we did not assess the impact of co-mutations, such as *STK11*/*LKB1* [34], on clinical outcomes. Third, in the case of longitudinal samples, we evaluated only three time-points; additional early- or later- time points [22] in a larger patient cohort, could strengthen the significance of our findings. Other interesting perspectives would be the assessment of *KRAS* MAF changes in cfDNA of NSCLC patients treated with the novel targeted therapies such as the *KRAS* G12C specific inhibitor and the application of next-generation sequencing to track other tumor-specific co-mutations with potential prognostic significance in plasma samples.

## 5. Conclusions

Our study shows that *KRAS* G12/G13 mutations in cfDNA assessed using ddPCR are significantly associated with poor clinical outcomes, in terms of disease control, PFS and OS among patients with NSCLC treated with first-line systemic treatment, delineating the prognostic value of plasma *KRAS* mutations in NSCLC. Most importantly, *KRAS* G12/G13 mutational load in plasma represents an independent predictor of poor prognosis in these patients. We demonstrated that the dynamic changes in plasma *KRAS* MAF during treatment are correlated with disease progression thus underscoring the monitoring potential of this circulating biomarker. Therefore, exploration of *KRAS* G12/G13 mutational load in cfDNA has great potential in assisting clinicians not only in predicting patients’ outcomes but also for treatment monitoring, towards the optimal management of NSCLC patients.

## Figures and Tables

**Table 1:** Clinicopathological characteristics of NSCLC patients included in the study.

Variable	No. of Patients (%)
TNM Stage ^a^	
III	24 (21.05)
IV	88 (77.19)
Unknown	2 (1.75)
Number of metastatic organs	
≤2	88 (77.19)
>2	26 (22.81)
Smoking history	
Non-smokers	7 (6.14)
Former smokers	33 (28.95)
Smokers	68 (59.65)
Unknown	6 (5.26)
PD-L1 status ^b^	
Negative	37 (32.46)
Positive	45 (39.47)
Unknown	32 (28.07)
Gender	
Males	92 (80.70)
Females	22 (19.30)
ECOG Performance status ^c^	
0	86 (75.44)
1	25 (21.93)
2	2 (1.75)
Unknown	1 (0.88)
Histological subtype	
Adenocarcinoma	91 (79.82)
Squamous cell carcinoma	15 (13.16)
Other	8 (7.02)
First-Line Treatment	
Chemotherapy	86 (75.44)
Combination chemo-immunotherapy	20 (17.54)
Immunotherapy alone	5 (4.39)
Unknown	3 (2.63)
*KRAS* tissue status ^d^	
Wild-Type	61 (53.51)
G12C	16 (14.04)
G12D	10 (8.77)
G12V	5 (4.39)
G13D	2 (1.75)
G12S	1 (0.88)
G12A	1 (0.88)
Unknown	18 (15.79)

^a^ Tumor Node Metastasis (TNM) staging system. ^b^ PD-L1 status was determined by immunohistochemistry. ^c^ Eastern Cooperative Oncology Group Performance Status. ^d^ Determined by Sanger sequencing.

**Table 2:** Descriptive statistics for continuous variables of the study in NSCLC patients.

				Percentiles		
Variable	No. of Patients	Mean ± S.E. ^a^	Range	25th	50th (Median)	75th
*KRAS* G12/G13 MAF ^b^						
MUT^c^ plasma samples	27	7.02 ± 1.49	0.2–28.27	1.53	3.95	10.04
*KRAS* G12/G13 copies/mL plasma						
MUT ^c^ plasma samples	27	1553.6 ± 729.38	19.70–18,600.0	62.50	192.0	1080.0
cfDNA concentration ^d^ (ng/μL)	114	15.88 ± 8.86	0.25–800.0	0.70	1.45	3.19
Patients’ Age (years)	114	65.59 ± 0.91	37.0–84.0	60.00	67.00	72.25
PFS (months)	114	4.97 ± 0.52	0.13–25.58	1.60	2.79	5.53
OS (months)	114	9.05 ± 0.92	0.13– 44.68	1.95	5.08	13.07

^a^ Standard error. ^b^ MAF, Mutant allele frequency. ^c^ MUT, *KRAS* G12/G13 mutant samples. ^d^ Concentration was determined using Qubit fluorometer 2.0 (Life Technologies).

**Table 3:** Associations of *KRAS* G12/G13 MAF in ctDNA with clinicopathological data of NSCLC patients.

Variable	No. of Patients	Mean ± S.E. ^a^	Range	*p*-Value
TNM Stage ^b^				0.213 ^c^
III	24	0.34 ± 0.17	0.0–3.89	
IV	88	2.06 ± 0.56	0.0–28.27	
Smoking status				
Non-smokers	7	0.05 ± 0.03	0.0–0.21	0.135 ^d^
Former smokers	33	3.07 ± 1.06	0.0–28.27	
Smokers	68	1.41 ± 0.53	0.0–26.14	
PD-L1 status				
Negative	37	1.01 ± 0.38	0.0–8.59	0.239 ^c^
Positive	45	2.23 ± 0.84	0.0–26.14	
Histologic type				
Adenocarcinoma	91	1.85 ± 0.46	0.0–26.14	0.183 ^d^
Squamous cell carcinoma	15	0.17 ± 0.06	0.0–0.67	
Other	8	3.54 ± 3.53	0.0–28.27	
Performance status ^e^				
0	86	1.29 ± 0.37	0.0–19.62	0.379 ^c^
1 or 2	27	3.27 ± 1.43	0.0–28.27	
Gender				
Males	92	1.38 ± 0.41	0.0–28.27	0.361 ^c^
Females	22	3.27 ± 1.49	0.0–26.14	
Number of metastatic organs				
≤2	88	1.30 ± 0.39	0.0–28.27	0.064 ^c^
>2	26	3.25 ± 1.38	0.0–26.14	

^a^ Standard error. ^b^ TNM staging system. ^c^ Calculated by the “Mann–Whitney U test”. ^d^ Calculated by the “Jonckheere-Terpstra Test”. ^e^ Eastern Cooperative Oncology Group Performance Status.

**Table 4:** Cox univariate regression analysis: prognostic performance of *KRAS* G12/G13 mutational load in ctDNA and clinicopathological variables of NSCLC patients.

	Univariate Analysis	
Variable	PFS	OS
*KRAS* ctDNA status (MUT vs. WT)		
HR ^a^	3.42	2.72
95% CI ^b^	1.92–6.07	1.55–4.77
*p*-value	**<0.001**	**<0.001**
Log_10_ *KRAS* G12/G13 MAF		
HR ^a^	1.97	2.04
95% CI ^b^	1.25–3.11	1.28–3.24
*p*-value	**0.00** **3**	**0.003**
Log_10_ *KRAS* G12/G13 copies/mL		
HR ^a^	1.76	1.65
95% CI ^b^	1.26–2.47	1.18–2.30
*p*-value	**0.00** **1**	**0.003**
TNM stage (IV vs. III)		
HR ^a^	2.13	2.44
95% CI ^b^	1.12–4.07	1.20–4.97
*p*-value	**0.0** **22**	**0.014**
Number of metastatic organs (>2 vs. ≤2)		
HR ^a^	2.49	4.24
95% CI ^b^	1.17–5.28	2.00–8.99
*p*-value	**0.018**	**<0.001**
Performance status (1 or 2 vs. 0)		
HR ^a^	1.89	1.78
95% CI ^b^	1.14–3.13	1.03–3.08
*p*-value	**0.013**	**0.041**
Histologic subtype (ADC vs. Other)		
HR ^a^	1.02	1.01
95% CI ^b^	0.57–1.82	0.54–1.90
*p*-value	0.950	0.964
Smoking history (Smokers vs. No-smokers)		
HR ^a^	0.67	0.919
95% CI ^b^	0.42–1.07	0.55–1.54
*p*-value	0.090	0.749
PD-L1 status (Positive vs. Negative)		
HR ^a^	0.79	0.787
95% CI ^b^	0.47–1.35	0.44–1.39
*p*-value	0.396	0.410
Gender (Female vs. Male)		
HR ^a^	0.885	1.11
95% CI ^b^	0.49–1.57	0.63–1.98
*p*-value	0.678	0.716

^a^ Hazard ratio, estimated from Cox proportional hazard regression model. ^b^ Confidence interval of the estimated HR. The values in bold indicate statistical significance (*p* < 0.05).

**Table 5:** Cox multivariate analysis of KRAS G12/G13 MAF as continuous and as dichotomous variable, regarding PFS and OS.

	PFS			OS		
Variable	HR ^a^	95% CI ^b^	*p*-Value	HR ^a^	95% CI ^b^	*p*-Value
Full multivariate Model						
*KRAS* G12/G13 status						
WT	1.00					
MUT	3.12	1.72–5.67	**<0.001**	2.53	1.40–4.56	**0.002**
TNM stage						
III	1.00					
IV	1.60	0.82–3.13	0.170	1.97	0.94–4.15	0.073
Metastatic organs						
≤2	1.00					
>2	1.95	0.91–4.23	0.089	3.38	1.56–7.34	**0.002**
Performance status						
0	1.00					
1 or 2	1.77	1.04–3.02	**0.035**	1.56	0.86–2.80	0.141
*KRAS* G12/G13 MAF						
Log_10_ (*KRAS* G12/G13 MAF)	1.99	1.25–3.16	**0.004**	1.85	1.21–2.85	0.005
TNM stage						
III	1.00					
IV	1.70	0.37–7.76	0.495	2.05	0.55–7.74	0.288
Metastatic organs						
≤2	1.00					
>2	1.32	0.48–3.63	0.590	2.88	1.07–7.80	**0.037**
Performance status						
0	1.00					
1 or 2	2.29	0.99–5.30	**0.030**	1.88	0.80–4.43	0.148
Reduced Multivariate Models						
*KRAS* G12/G13 status ^c^						
MUT	3.05	1.69–5.48	**<0.001**	2.70	1.51–4.82	**0.001**
*KRAS* G12/G13 MAF ^c^						
Log_10_ (*KRAS* G12/G13 MAF)	1.82	1.16–2.86	**0.009**	1.93	1.23–3.03	**0.004**
*KRAS* G12/G13 status ^d^						
MUT	3.20	1.79–5.73	**<0.001**	2.48	1.40–4.39	**0.002**
*KRAS* G12/G13 MAF ^d^						
Log_10_ (*KRAS* G12/G13 MAF)	1.92	1.22–3.04	**0.005**	1.95	1.23–3.08	**0.004**
*KRAS* G12/G13 status ^e^						
MUT	3.55	1.98–6.36	**<0.001**	2.73	1.55–4.80	**0.001**
*KRAS* G12/G13 MAF ^e^						
Log_10_ (*KRAS* G12/G13 MAF)	2.08	1.32–3.28	**0.002**	1.89	1.21–2.95	**0.005**

^a^ Hazard ratio, estimated from Cox proportional hazard regression model. ^b^ Confidence interval of the estimated HR. ^c^ Reduced multivariate model adjusted for TNM stage. ^d^ Reduced multivariate model adjusted for number of metastatic organs. ^e^ Reduced multivariate adjusted for patients’ performance status. The values in bold indicate statistical significance (*p* < 0.05).

## Supplementary Materials

The following are available online at https://www.mdpi.com/2073-4409/9/11/2514/s1, Figure S1: Kaplan-Meier survival curves according to tissue *KRAS* mutation subtypes, Figure S2: Kaplan-Meier survival curves according to *KRAS* G12/G13 mutational status in cfDNA and paired tissue samples, Table S1: Concordance in *KRAS* G12/G13 mutation status as determined using Sanger sequencing in tumor-tissues and via ddPCR in plasma ctDNA in 96 paired samples.
