# Supplementary material for: Detection of KRAS G12/G13 Mutations in Cell Free-DNA by Droplet Digital PCR, Offers Prognostic Information for Patients with Advanced Non-Small Cell Lung Cancer
Source: Cells. 2020 Nov 20;9(11):2514. doi: 10.3390/cells9112514 (PMC7699710; doi:10.3390/cells9112514)
Supplement: Supplementary file 1 [file cells-09-02514-s001.zip › Supplementary Table 1.docx]

**Supplementary Table 1:** Concordance in *KRAS* G12/G13 mutation status as determined using Sanger sequencing in tumor-tissues and *via* ddPCR in plasma ctDNA in 96 paired samples

|  | **Plasma cfDNA analysis^a^** | |  |
| --- | --- | --- | --- |
| **Tumor-tissue analysis^b^** | **pWT (%)^c^** | **pMUT (%)^c^** | Total, N |
| tWT | 54 (88.5) | 7 (11.5) | 61 |
| tMUT | 17 (48.6) | 18 (51.4) | 35 |
| Total, N | 71 | 25 | 96 |
| Overall concordance | N=72/96 (75.0%), kappa^d^ 0.395 (95% CI, 0.20–0.59; *P*<0.001) | | |
| **Tumor-tissue analysis^b^** | **pWT (%)^c^** | **pMUT (%)^c^** | Total, N |
| G12C | 10 (62.5) | 6 (37.5) | 16 |
| G12D | 6 (60.0) | 4 (40.0) | 10 |
| G12S | 0 (0.0) | 1 (100.0) | 1 |
| G12V | 1 (20.0) | 4 (80.0) | 5 |
| G12A | 0 (0.0) | 1 (100.0) | 1 |
| G13D | 0 (0.0) | 2 (100.0) | 2 |
| Total, N | 17 | 18 | 35 |

***^a^*** *KRAS G12/G13 status in plasma as determined using ddPCR*

*^b^ KRAS G12/G13 mutations in tumor tissues were assessed using Sanger sequencing*

*^c^ Percentages are calculated using tissue KRAS mutation status as a reference*

*^d^ Cohen's kappa coefficient*
