# Supplementary figures and images for: Detection of KRAS G12/G13 Mutations in Cell Free-DNA by Droplet Digital PCR, Offers Prognostic Information for Patients with Advanced Non-Small Cell Lung Cancer
Source: Cells. 2020 Nov 20;9(11):2514. doi: 10.3390/cells9112514 (PMC7699710; doi:10.3390/cells9112514)

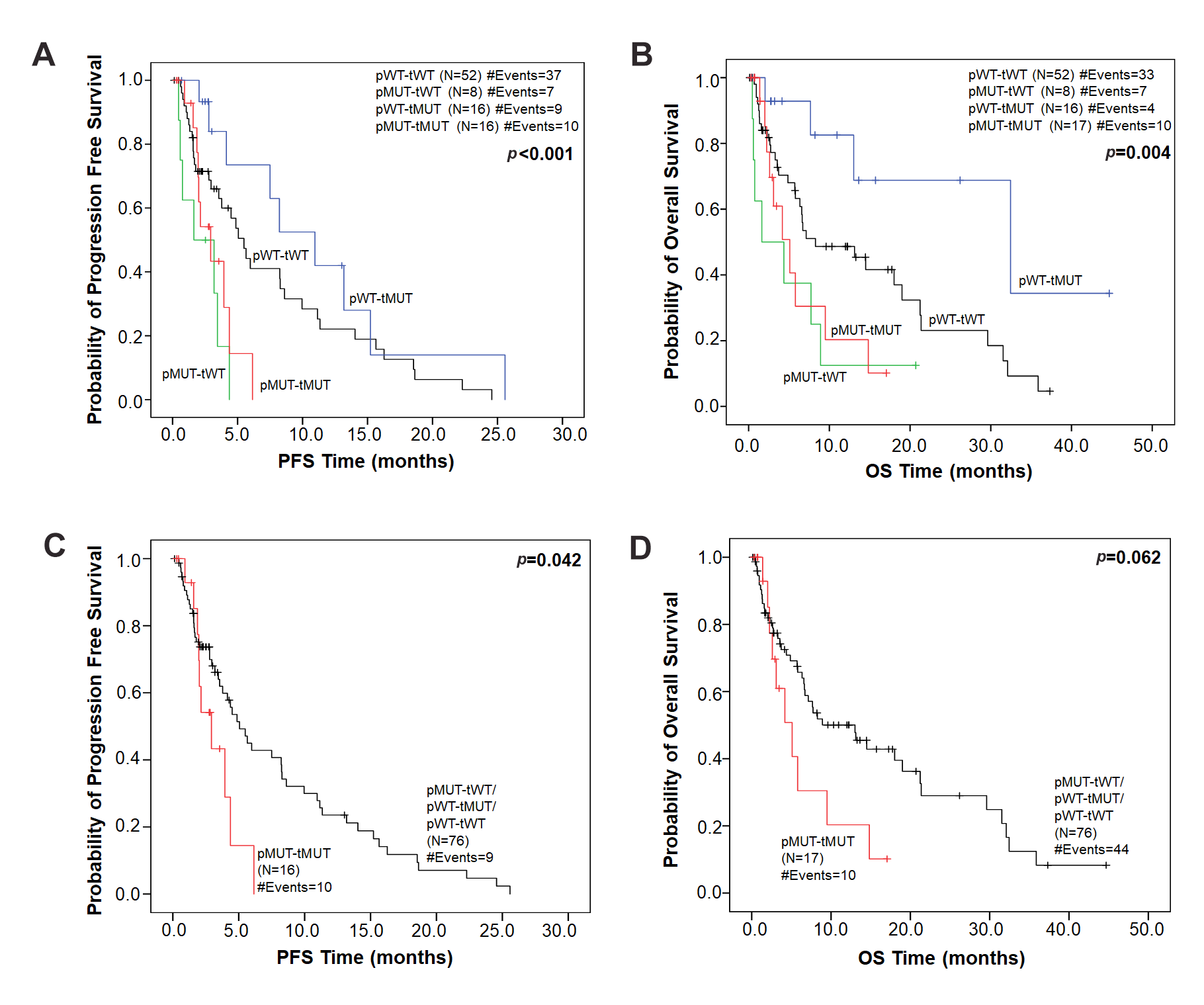

Supplement: Supplementary file 1 [file cells-09-02514-s001.zip › Supplementary Figure 2.tif]

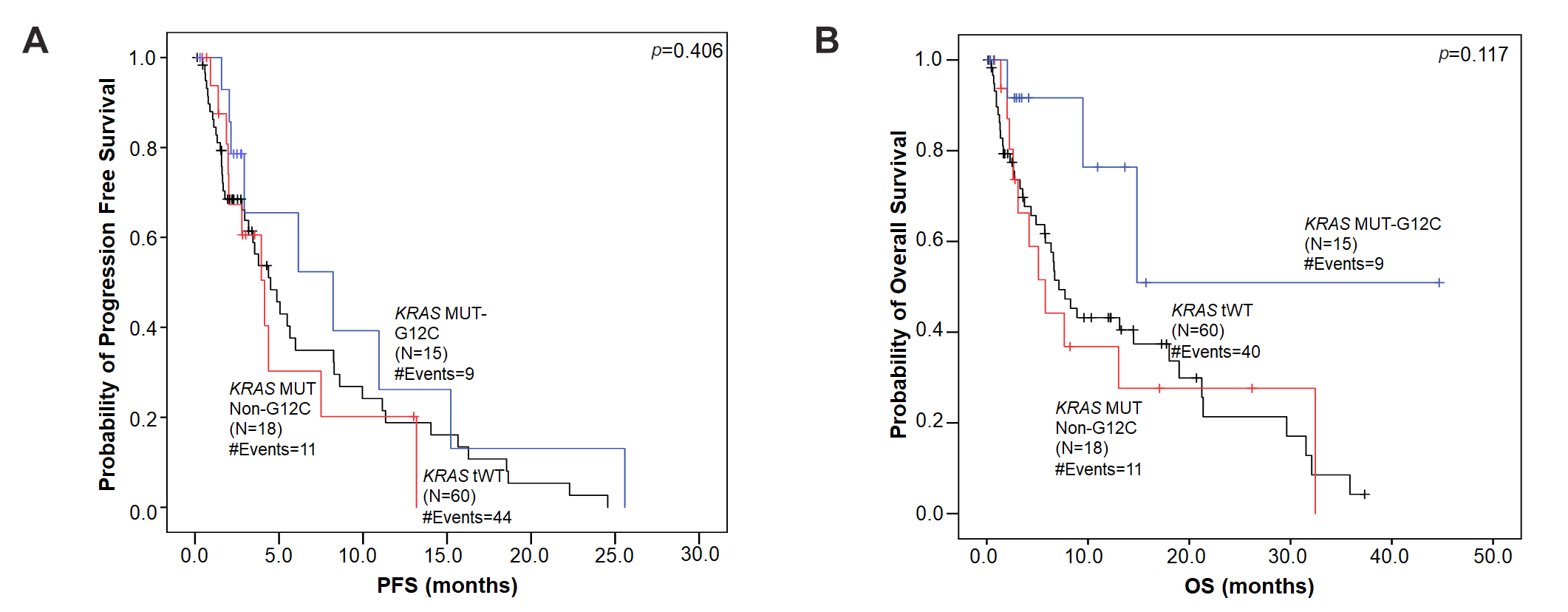

Supplement: Supplementary file 1 [file cells-09-02514-s001.zip › Supplementary Figure 1.tif]
